# The Effect of Alcohol and Hydrogen Peroxide on Liver Hepcidin Gene Expression in Mice Lacking Antioxidant Enzymes, Glutathione Peroxidase-1 or Catalase

**DOI:** 10.3390/biom5020793

**Published:** 2015-05-06

**Authors:** Duygu Dee Harrison-Findik, Sizhao Lu

**Affiliations:** 1 Department of Internal Medicine, Division of Gastroenterology/Hepatology, University of Nebraska Medical Center, Omaha, NE 68198, USA; 2 Department of Biochemistry and Molecular Biology, University of Nebraska Medical Center, Omaha, NE 68198, USA; E-Mail: sizhao.lu@unmc.edu

**Keywords:** alcoholic liver disease, CHOP, endoplasmic reticulum stress, *Hamp*, hepatocyte, iron, oxidative stress

## Abstract

This study investigates the regulation of hepcidin, the key iron-regulatory molecule, by alcohol and hydrogen peroxide (H_2_O_2_) in glutathione peroxidase-1 (gpx-1^−/−^) and catalase (catalase^−/−^) knockout mice. For alcohol studies, 10% ethanol was administered in the drinking water for 7 days. Gpx-1^−/−^ displayed significantly higher hepatic H_2_O_2_ levels than catalase^−/−^ compared to wild-type mice, as measured by 2'-7'-dichlorodihydrofluorescein diacetate (DCFH-DA). The basal level of liver hepcidin expression was attenuated in gpx-1^−/−^ mice. Alcohol increased H_2_O_2_ production in catalase^−/−^ and wild-type, but not gpx-1^−/−^, mice. Hepcidin expression was inhibited in alcohol-fed catalase^−/−^ and wild-type mice. In contrast, alcohol elevated hepcidin expression in gpx-1^−/−^ mice. Gpx-1^−/−^ mice also displayed higher level of basal liver CHOP protein expression than catalase^−/−^ mice. Alcohol induced CHOP and to a lesser extent GRP78/BiP expression, but not XBP1 splicing or binding of CREBH to hepcidin gene promoter, in gpx-1^−/−^ mice. The up-regulation of hepatic ATF4 mRNA levels, which was observed in gpx-1^−/−^ mice, was attenuated by alcohol. In conclusion, our findings strongly suggest that H_2_O_2_ inhibits hepcidin expression *in vivo*. Synergistic induction of CHOP by alcohol and H_2_O_2,_ in the absence of gpx-1, stimulates liver hepcidin gene expression by ER stress independent of CREBH.

## 1. Introduction

Patients with alcoholic liver disease (ALD) frequently display increased levels of iron and even moderate alcohol intake affects iron homeostasis [1,2]. Hepcidin, a circulatory peptide synthesized in the liver, is the key iron-regulatory hormone, which controls iron absorption through the duodenum and the release of iron from macrophages [3]. A role for hepcidin has been suggested in ALD [4,5,6,7]. We have reported that oxidative stress induced by short-term alcohol-intake is sufficient to suppress liver hepcidin expression by inhibiting the activity of transcription factor C/EBP alpha (C/EBPα) [8]. The suppression of hepatic hepcidin expression by alcohol results in elevated expression of intestinal iron transporters, which is abolished by vitamin E treatment of mice [8].

Ethanol metabolism in the liver produces reactive oxygen species (ROS) including superoxide (O_2_^•−^) [9]. O_2_^•−^ is short-lived and rapidly converted to H_2_O_2_ by superoxide dismutase enzymes, SOD1 and SOD2. H_2_O_2_ can react with iron to form the highly reactive and damaging hydroxyl radicals [10]. The removal of excess H_2_O_2_ and the tight regulation of iron metabolism are therefore important to prevent tissue injury. The antioxidant enzymes, glutathione peroxidase (gpx), catalase and peroxiredoxins catabolize H_2_O_2_ [11,12]. Gpx-1 is the most abundant and ubiquitously expressed member of gpx family [13]. Catalase is localized to peroxisomes whereas gpx-1 is present in both cytosol and mitochondria [13]. The protein and activity levels of gpx-1 and catalase have been reported to be significantly reduced in rats livers following intragastric administration of alcohol and fish oil [14]. A decrease in both cytosolic and mitochondrial gpx-1 activity in the liver has also been shown in rats fed with liquid alcohol diets [15].

The activation of unfolding protein response (UPR) signaling is an adaptive mechanism in response to oxidative stress [16]. UPR and endoplasmic reticulum (ER) stress are known to play a role in the pathogenesis of ALD [17,18]. H_2_O_2_ synergizes with low doses of alcohol to induce UPR and ER stress in a zebra fish model of ALD [19]. UPR-responsive transcription factor, ATF4 (activating transcription factor-4) plays a role in redox regulation [18,20]. ATF4 is also the dominant transactivator of transcription factor, C/EBP homologous protein (CHOP, a.k.a GADD153) [21]. CHOP can inhibit the function of C/EBP family of transcription factors including C/EBPα by forming heterodimers [22]. CHOP is also involved in the regulation of cellular redox state and apoptosis [23]. Two independent studies have shown a role for ER stress in hepcidin induction either via the CHOP/CEBPα axis or by the direct binding of the transcription factor, cyclic AMP response element binding protein-H (CREBH) to hepcidin gene promoter [24,25]. Studies using tissue culture cells suggested both induction and inhibition of hepcidin by H_2_O_2_ *in vitro* [26,27].

The roles of H_2_O_2_ and/or ER stress in hepcidin regulation by alcohol metabolism *in vivo* are unknown. Understanding these mechanisms is important because iron and alcohol act synergistically to induce liver injury. This study addresses these questions by employing mice with or without impaired H_2_O_2_ catabolism and subjecting them to alcohol exposure in an experimental model.

## 2. Results and Discussion

### 2.1. The Effect of Alcohol on H_2_O_2_ Production and Hepcidin Expression in Catalase^−/−^ and Gpx-1^−/−^ Mice

In order to study the synergistic action of alcohol and hydrogen peroxide (H_2_O_2_), transgenic mice, lacking the expression of antioxidant enzymes, glutathione peroxidase-1 (gpx-1^−/−^) or catalase (catalase^−/−^) on both alleles, were fed with plain water (control) or ethanol for 1 week. This feeding protocol did not alter liver histology or serum ALT levels, and the body weights were similar between experimental groups at the beginning and end of the 7 day period, as reported previously [7,8]. Both wild-type and transgenic mice consumed around 4 mL of 10% ethanol or 5 mL of plain water per day. The blood alcohol levels in wild-type, catalase^−/−^ and gpx-1^−/−^ mice were similar and matched the values we have published previously with 129/Sv strain mice [8] (Table 1). 7 day-long alcohol feeding induced a weak but significant increase in CYP2E1 activity, which was similar in wild-type and both knockout mice (Table 1). These findings suggest that alcohol exposure and/or the deficiency of catalase or gpx-1 does not induce liver injury in our experimental model.

**Table 1:** The levels of blood alcohol and liver CYP2E1 enzyme activity in wild-type and knockout mice were measured, as described in the experimental section. (N.D. = not detectable).

Mouse identity	Blood alcohol (mg/dL)	CYP2E1 activity (*nmole 4-nc/h/mg protein*)
Wild-type water-fed	N.D.	57 ± 3
Wild-type alcohol-fed	123 ± 16	92 ± 3.9
Catalase^−/−^ water-fed	N.D.	61 ± 6
Catalase^−/−^ alcohol-fed	125 ± 13	100 ± 2
Gpx-1^−/−^ water-fed	N.D.	62 ± 4
Gpx-1^−/−^ alcohol-fed	127 ± 14	100 ± 3

**Figure 1:**
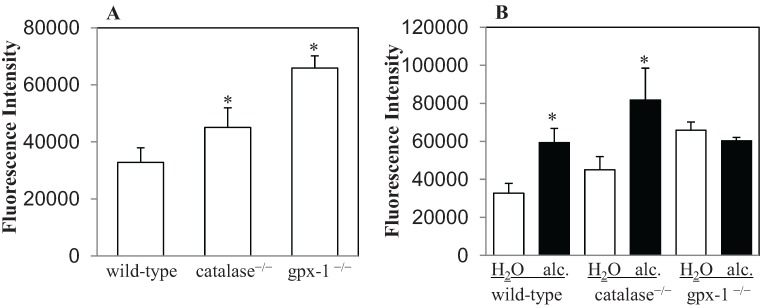
Intracellular H_2_O_2_ levels in hepatocytes freshly isolated by perfusion from the livers of untreated wild-type and catalase^−/−^ or gpx-1^−/−^ transgenic mice (**A**), and mice fed with 10% ethanol (alc.) or plain water (H_2_O) for 1 week (**B**) were measured by 2'-7'-dichlorodihydrofluorescein (DCF) fluorescence. DCF fluorescence detected by spectrophotometer in cells incubated with 2'-7'-dichlorodihydrofluorescein diacetate (DCFH-DA) was normalized to that in cells incubated with 0.1% DMSO (control), and expressed as arbitrary fluorescence per 0.5 × 10^6^ hepatocytes. Asterisks indicate statistical significance (*p* < 0.05).

The effect of alcohol exposure on H_2_O_2_ production was determined by 2'-7'-dichlorodihydrofluorescein diacetate (DCFH-DA) assays. For these experiments, hepatocytes, freshly isolated by perfusion from the livers of control and ethanol-fed mice, were incubated with DCFH-DA, as described in Experimental Section. After DCFH-DA enters the cell, it is deacetylated to DCFH by intracellular esterases, and then oxidized by peroxides to highly fluorescent 2'-7'-dichlorodihydrofluorescein (DCF) [28]. Significantly higher levels of DCF fluorescence were observed in the hepatocytes of untreated catalase^−/−^ and gpx-1^−/−^ mice than in untreated wild-type mice (Figure 1A). Gpx-1^−/−^ mice exhibited the most prominent hepatic H_2_O_2_ accumulation. DFC fluorescence in gpx-1^−/−^ mice was 2-fold higher than wild-type mice (Figure 1A). Similarly, the level of fluorescence in gpx-1^−/−^ was also significantly greater than that in catalase^−/−^ mice (Figure 1A). In contrast, alcohol exposure elevated hepatic H_2_O_2_ levels in wild-type and catalase^−/−^, but not in gpx-1^−/−^, mice compared to their water-fed counterparts (Figure 1B). Alcohol-induced increase in hepatic H_2_O_2_ content was observed most significantly in catalase^−/−^ mice. Untreated or alcohol-fed wild-type and gpx-1^−/−^ mice exhibited significantly less fluorescence intensity than alcohol-fed catalase^−/−^ mice (Figure 1B). Furthermore, the amount of hepatic H_2_O_2_ in alcohol-fed wild-type mice was similar to that in gpx-1^−/−^ mice (Figure 1B).

**Figure 2:**
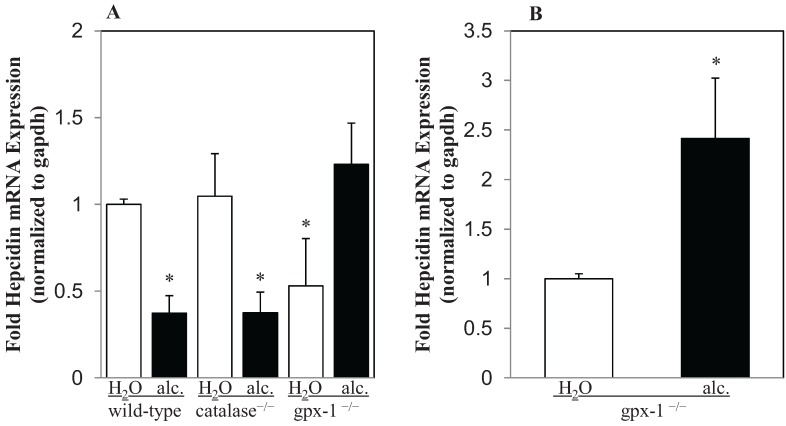
Hepcidin mRNA expression in the liver. cDNA synthesized from liver RNA of transgenic mice, lacking the expression of either glutathione peroxidase-1 (gpx-1^−/−^) or catalase (catalase^−/−^), and wild-type mice, fed with plain water (H_2_O) or ethanol (alc.), was employed to determine hepcidin mRNA expression by Taqman real-time PCR. (**A**) Hepcidin gene expression in water or alcohol-fed transgenic mice and alcohol-fed wild-type mice was expressed as-fold hepcidin expression of that in wild-type mice fed with water. (**B**) Hepcidin gene expression in alcohol-fed gpx-1^−/−^ mice was expressed as-fold hepcidin expression of that in gpx-1^−/−^ mice fed with water. Asterisks indicate statistical significance (*p* < 0.05).

The combined effect of alcohol and H_2_O_2_ in the regulation of hepcidin gene expression was determined by real-time PCR, as described in Experimental Section. Alcohol inhibited hepcidin mRNA expression in the livers of wild-type mice (Figure 2A). The deletion of catalase gene in catalase^−/−^ transgenic mice did not significantly alter the basal level of hepcidin expression in the liver (Figure 2A). Similar to wild-type mice, hepcidin expression in the livers of catalase^−/−^ mice was also significantly inhibited by alcohol (Figure 2A). Contrary to catalase^−/−^, the basal level of hepcidin mRNA expression was decreased by two-fold in gpx-1^−/−^ mice compared to wild-type mice (Figure 2A). Alcohol however up-regulated hepcidin gene expression in gpx-1^−/−^ mice over two-fold compared to that in water-fed gpx-1^−/−^ mice (Figure 2B).

Our knockout mice studies indicated a correlation between H_2_O_2_ and the inhibition of hepcidin expression in the liver. Namely, H_2_O_2_ accumulation in the livers of untreated gpx-1^−/−^ and alcohol-fed wild-type or catalase^−/−^ mice was accompanied by a significant decrease in hepcidin expression. This inhibition might be concentration-dependent because the level of hepatic H_2_O_2_ in untreated catalase^−/−^ mice, which was significantly lower than that in untreated gpx-1^−/−^, was not sufficient to inhibit hepcidin. Miura *et al*. [26] and Millonig *et al*. [27] have reported the regulation of hepcidin expression by H_2_O_2_ in tissue culture cells. Millonig *et al*. have shown an induction and inhibition of hepcidin expression by low and high concentrations of H_2_O_2_, respectively. They suggested the involvement of Stat3 in the stimulation and cytotoxicity in the inhibition of hepcidin expression by H_2_O_2_ *in vitro* [27]. Miura *et al*. however have reported a role for elevated histone deacetylase activity in H_2_O_2_-mediated inhibition of hepatic hepcidin in Huh7 cells [26]. Although antioxidant defense mechanisms in the liver *in vivo* are more elaborate than in cultured hepatoma cells, these studies show a concentration-dependent effect of H_2_O_2_ on liver hepcidin expression. Given the fact that the intracellular localization of catalase and gpx-1 are not similar, our study also pointed out the spatial dynamics of H_2_O_2_ in hepcidin regulation. Namely, the regulation of hepcidin expression was different in catalase^−/−^ and gpx-1^−/−^ mice. Unlike catalase, which is primarily expressed in peroxisomes, gpx-1 is expressed both in cytosol and mitochondria [13]. We have previously reported that mitochondrial superoxide is not involved in inhibition of hepcidin expression by alcohol [29]. Attenuation of hepcidin expression in untreated gpx-1^−/−^ mice, which displayed a higher H_2_O_2_ content than untreated catalase^−/−^ mice, suggested that cytosolic and mitochondrial H_2_O_2_ might be involved in inhibition of hepcidin expression. However, upon alcohol exposure, gpx-1^−/−^ mice displayed induction of hepcidin expression without changes in H_2_O_2_ levels. Nevertheless, our knockout mice studies suggested that both subcellular location and concentration of H_2_O_2_ in hepatocytes are equally important in differential regulation of hepcidin expression.

### 2.2. The Effect of Alcohol and H_2_O_2_ on ER Stress in the Liver

Elevated hepcidin expression in alcohol-fed gpx-1^−/−^ mice may involve various mechanisms such as inflammation or ER stress, which are known to induce hepcidin expression [24,25]. Our previous studies have however shown that alcohol can inhibit liver hepcidin expression in the presence of inflammation [30,31]. We have also shown the involvement of alcohol-mediated TLR4 and NF-κB activation in this process [31]. It is therefore not feasible that the induction of hepcidin in alcohol-treated gpx-1^−/−^ mice is mediated by inflammation. We then studied the role of ER stress in this process.

**Figure 3:**
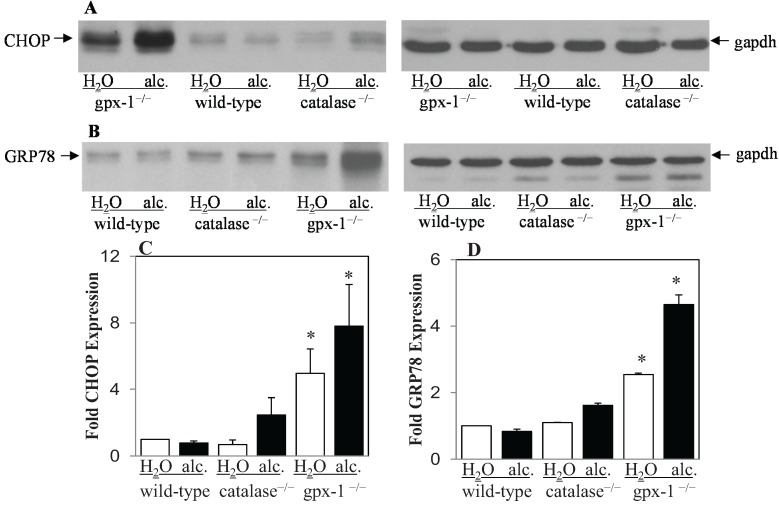
Liver CHOP (GADD153) and GRP78 (BiP) protein expression. Total cell lysates isolated from the livers of H_2_O or ethanol (alc.)-fed wild-type and gpx-1^−/−^ or catalase^−/−^ transgenic mice were employed to detect CHOP (**A**) or GRP78/BiP (**B**) protein expression by western blotting. An anti-gapdh antibody was used to demonstrate equal protein loading. CHOP (**C**) and GRP78 (**D**) expression was quantified by densitometric analysis and normalized to gapdh expression, respectively. Normalized expression in H_2_O or alcohol-fed knockout and alcohol-fed wild-type mice was expressed as fold expression of that in H_2_O-fed wild-type mice. Asterisks indicate statistical significance (*p* < 0.05).

Iron-regulatory genes have been reported to be the downstream targets of the transcription factor, CHOP (GADD153), which is involved in ER stress [24,32]. In untreated cells, CHOP is expressed at very low levels and its expression is induced by stress [21]. We therefore determined the expression level of CHOP in the livers of untreated and alcohol-treated wild-type, gpx-1^−/−^ and catalase^−/−^ mice by western blotting (Figure 3A). CHOP protein expression was significantly elevated in the livers of untreated gpx-1^−/−^, but not catalase^−/−^ mice, compared to untreated wild-type mice. Alcohol induced liver CHOP expression strongly in gpx-1^−/−^, and only marginally and not significantly in catalase^−/−^ mice, compared to wild-type mice (Figure 3A,C). To further examine ER stress, the expression of chaperone protein, GRP78 (BiP) in the livers of untreated and alcohol-treated wild-type, catalase^−/−^ and gpx-1^−/−^ mice was detected by western blotting (Figure 3B). GRP78 protein expression was significantly elevated in the livers of untreated gpx-1, but not catalase, knockout mice, compared to untreated wild-type mice. Alcohol treatment further induced liver GRP78 expression significantly in gpx-1^−/−^, but not catalase^−/−^ or wild-type, mice (Figure 3B,D).

**Figure 4:**
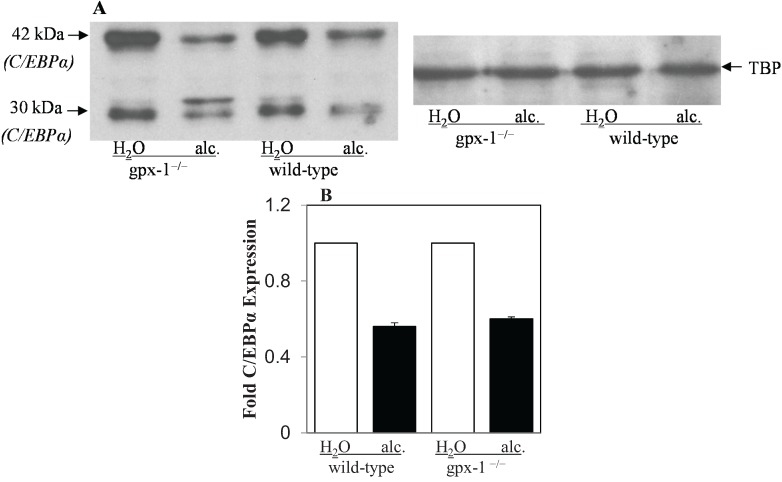
Liver C/EBPα protein expression. (**A)** Nuclear cell lysates isolated from the livers of H_2_O or ethanol (alc.)-fed wild-type and gpx-1^−/−^ transgenic mice were employed to detect C/EBPα protein expression by western blotting. An anti-TATA-binding protein (TBP) antibody was used to demonstrate equal nuclear protein loading. (**B**) C/EBPα protein (30 kDa and 42 kDa) expression was quantified by densitometric analysis and normalized to TBP expression. Normalized expression in H_2_O or alcohol-fed gpx-1^−/−^ and alcohol-fed wild-type mice was expressed as fold expression of that in H_2_O-fed wild-type mice.

CHOP acts as a negative regulator of the C/EBP family of transcription factors. We have previously shown that alcohol inhibits C/EBPα in wild-type mice livers [8]. Since CHOP was induced by alcohol in gpx-1 mice, we determined nuclear C/EBPα expression in gpx-1^−/−^ mice livers by western blotting. The absence of gpx-1 did not significantly alter the basal expression level of liver C/EBPα protein in gpx-1^−/−^ mice compared to wild-type mice (Figure 4A,B). Alcohol inhibited C/EBPα expression to the same extent in gpx-1^−/−^ and wild-type mice (Figure 4A,B).

**Scheme 1:**
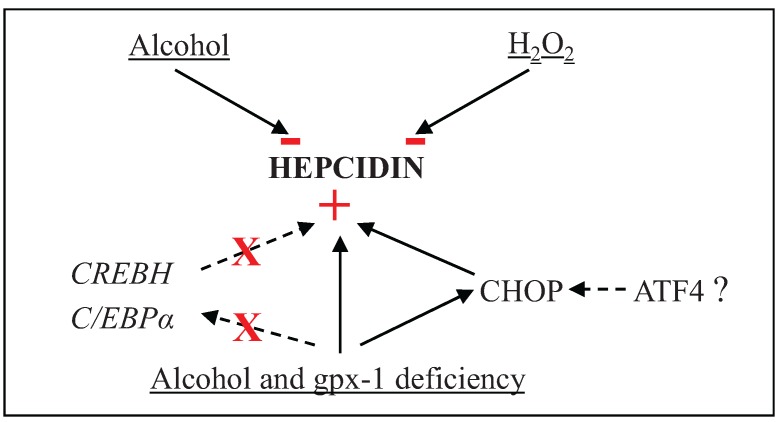
H_2_O_2_ and/or alcohol-mediated regulation of hepatic hepcidin gene expression. The signs, − and + indicate inhibition and induction of gene expression, respectively, and X represents the lack of CREBH or C/EBPα involvement in the induction of hepatic hepcidin gene expression.

The up-regulation of CHOP and Grp78 protein expression in the livers of gpx-1, but not catalase, knockout mice, suggest that accumulation of H_2_O_2_ in cytosol and mitochondria of hepatocytes together with alcohol metabolism can cause ER stress, thereby leading to the induction of hepcidin gene expression. C/EBPα, which is known to be inhibited by CHOP, is involved in the regulation of hepcidin gene expression [22,33]. We have previously shown that alcohol suppresses liver hepcidin expression via the inhibition of C/EBPα [7,8]. Oliveira *et al.* have also suggested that ER-stress-mediated biphasic regulation of hepcidin *in vitro* involves CHOP and C/EBPα [24]. However, our western blot analysis did not establish a correlation between CHOP and C/EBPα protein expression patterns in the livers of alcohol-fed mice. Namely, independent of differences in CHOP expression levels, both wild-type and gpx-1^−/−^ mice displayed inhibition of nuclear C/EBPα expression upon alcohol exposure. Based on these data and previous studies by us and others, we believe that CHOP does not associate with C/EBPα in alcohol-mediated induction of hepcidin expression in gpx-1 knockout mice (see Scheme 1 below). Similarly, Miura *et al*. have also shown that inhibition of C/EBPα by hepatitis C viral proteins does not involve CHOP [26]. Our findings nonetheless suggest that ER stress signaling may be involved in induction of liver hepcidin expression by alcohol in gpx-1^−/−^ mice.

**Figure 5:**
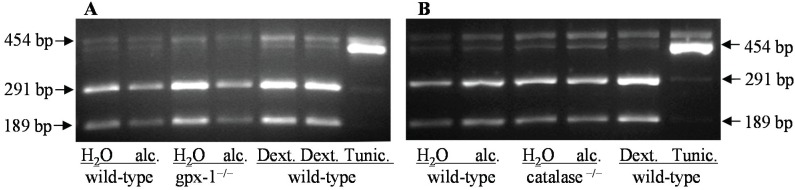
XBP1 mRNA splicing in the livers of H_2_O or ethanol (alc.)-fed wild-type and catalase^−/−^ or gpx-1^−/−^ transgenic mice was determined by RT-PCR and Pst1 restriction enzyme digestion. Wild-type mice injected with dextrose as control (Dext.) or tunicamycin (Tunic.) were used as controls. 454 bp, and 291bp and 189 bp amplicons refer to spliced (Pst1-resistant) and unspliced XBP1, respectively.

The effect of H_2_O_2_ on ER stress was further analyzed by determining the splicing of transcription factor, X-box binding protein 1 (XBP1), as described in Experimental Section (Figure 5). XBP1 is a specific substrate of the endoribonuclease, inositol-requiring enzyme 1 (IRE1). XBP1 mRNA splicing is therefore used as a marker for IRE1 activation. Splicing of XBP1 alters Pst1 recognition sequence present within the IRE1 excision site, and spliced XBP1 becomes resistant to Pst1 digestion. XBP1 was not spliced in the livers of untreated or alcohol-fed catalase^−/−^, gpx-1^−/−^ and wild-type mice, as confirmed by the presence of Pst1 digested 291 and 189 bp XBP1 DNA fragments (Figure 5A,B). In contrast, XBP1 was spliced in the livers of wild-type mice injected with an ER-inducer, tunicamycin, but not with dextrose (as control), as shown by the presence of a Pst1-resistant 454 bp XBP1 amplicon, and thereby validating our XBP1 splicing assay (Figure 5A,B). These findings show that unlike tunicamycin, alcohol and/or H_2_O_2_ did not activate the unfolded protein response transducer, IRE1 in the liver.

**Figure 6:**
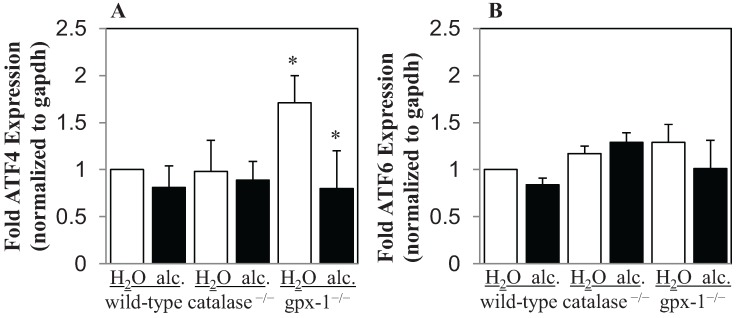
Liver ATF4 and ATF6 mRNA expression. cDNA synthesized from liver RNA of wild-type and catalase^−/−^ or gpx-1^−/−^ transgenic mice, fed with plain water (H_2_O) or ethanol (alc.), was employed to determine ATF4 (**A**) and ATF6 (**B**) mRNA expression by real-time PCR. Gene expression levels in alcohol fed wild-type and untreated or alcohol-fed transgenic mice were expressed as-fold ATF expression of that in wild-type mice fed with water. Asterisks indicate statistical significance (*p* < 0.05).

The transcription factors, ATF4 and ATF6, which are activated by ER stress, have been shown to regulate CHOP expression [21]. The level of ATF4 and ATF6 mRNA expression in the livers of untreated and alcohol-treated transgenic and wild-type mice was determined by real-time PCR (Figure 6). The expression of ATF4 in the liver was significantly increased in gpx-1^−/−^ and was unchanged in catalase^−/−^ mice, compared to wild-type mice (Figure 6A). Alcohol however significantly inhibited ATF4 mRNA expression in gpx-1^−/−^, but not catalase^−/−^ or wild-type, mice (Figure 6A). No significant changes in ATF6 mRNA expression were observed in transgenic mice compared to wild-type mice (Figure 6B). Similarly, ATF6 mRNA expression was not altered by alcohol treatment in wild-type or transgenic mice (Figure 6B).

**Figure 7:**
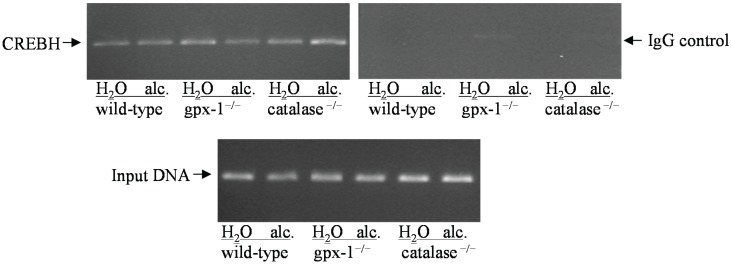
CREBH binding to hepcidin gene promoter. Chromatin isolated from the livers of water (H_2_O) or ethanol (alc.)-fed wild-type mice and gpx-1^−/−^ or catalase^−/−^ transgenic mice were immunoprecipitated by an anti-CREBH antibody or normal rabbit IgG, as control. The co-immunoprecipitated and total input (control) DNA were used as templates in PCR to amplify a 321 bp mouse hepcidin gene promoter region, which harbors CREBH DNA-binding site, as described in Experimental Section.

The transcription factor, cyclic AMP-responsive element binding protein H (CREBH) has been shown to be involved in ER stress-mediated regulation of hepcidin transcription. To study the effect of alcohol and/or H_2_O_2_ on hepcidin gene promoter, chromatin immunoprecipitation (CHIP) assays were performed, as described in Experimental Section. The occupancy of mouse hepcidin gene promoter by CREBH in the livers of untreated and alcohol-fed gpx-1^−/−^, catalase^−/−^ and wild-type mice was determined (Figure 7). Alcohol and/or H_2_O_2_ did not stimulate the binding of CREBH to hepcidin gene promoter in the livers of transgenic or wild-type mice (Figure 7). The amplification of total input DNA was similar in all the samples confirming that different liver chromatins contained equal amounts of DNA (Figure 7).

The elevation of ER stress is characterized by the activation of three distinct signaling pathways mediated by IRE1, ATF6 and PERK/eIF2α [34]. However, the lack of XBP1 splicing in the livers of knockout and wild-type mice suggests that the IRE1 arm of ER stress signaling is not activated by alcohol in these mice. Transcription factors, ATF4 and ATF6 are involved in the regulation of CHOP expression [21]. The expression of ATF4, but not ATF6, was elevated in gpx-1^−/−^ mice livers. It is therefore feasible that CHOP induction in untreated gpx-1^−/−^ mice is regulated by ATF4. However, alcohol inhibited ATF4 expression but induced CHOP expression in gpx-1^−/−^ mice suggesting the presence of other mechanisms. Different stress conditions have been shown to induce variable ATF4 expression [35]. Furthermore, ATF4 and CHOP are regulated at both the transcriptional and posttranscriptional levels [35,36]. The transcription factor, CREBH has also been reported to play a role in hepcidin up-regulation by tunicamycin-mediated ER stress [25]. The binding of CREBH to hepcidin gene promoter in gpx-1^−/−^, catalase^−/−^ or wild-type mice was however not stimulated by alcohol exposure, as shown by our CHIP assays. Compared to experimental ER stress models, other transcription factors besides CREBH may play a role in the regulation of hepcidin gene expression by ER stress in liver diseases. Future studies will investigate the mechanisms involved in synergistic regulation of CHOP by alcohol and H_2_O_2_.

## 3. Experimental Section

### 3.1. Animal Experiments

Animal experiments were approved by the animal ethics committee at the University of Nebraska Medical Center. Transgenic mice, homozygous for the null allele of glutathione peroxidase-1 (gpx-1^−/−^) or catalase (catalase^−/−^), on C57BL/6 genetic background, were generated, as described previously [37,38]. For alcohol treatment, male transgenic and wild-type C57BL/6 mice housed individually and maintained on rodent chow diet-7012 (*Harlan Teklad*), were administered 10% ethanol in the drinking water or plain water for 7 days, as described previously [7]. These experiments were performed 6 times with 2 mice in each group. Blood alcohol levels were measured by using a diagnostic alcohol kit (Sigma Aldrich Inc., St. Louis, MO, USA; product no. N7160), as published previously [8], and expressed as mg alcohol per dL blood.

### 3.2. Cytochrome P4502E1 (CYP2E1) Activity

CYP2E1 activity was measured in crude liver homogenates by the hydroxylation of p-nitrophenol to form 4-nitrocatechol (4-nc), as published previously [39,40].

### 3.3. Liver Perfusion

To isolate viable hepatocytes, mice livers were perfused, as described [29,41]. Briefly, livers were perfused (7 mL/min) with warm and gassed KRH buffer (25 mM HEPES, 114.9 mM NaCl, 4.5 mM KCl, 1 mM KH_2_PO_4_, 0.5 mM EGTA, pH 7.6) followed by KRH buffer containing 2mM Ca^2+^ and collagenase (0.214 mg/mL, Sigma C5138). Hepatocytes were washed thrice with ice-cold KRH buffer containing 2 mM Ca^2+^ and 2% BSA. Hepatocyte viability was ≥ 80%, as determined by Trypan Blue staining.

### 3.4. Measurement of Intracellular H_2_O_2_ Levels

Hepatocytes, freshly isolated by liver perfusion, were washed twice in KRH buffer containing 2 mM Ca^2+^. 0.5 × 10^6^ hepatocytes were incubated with 15 µM of the fluorescent probe, 2',7'-dichlorodihydrofluorescein-diacetate (DCFH-DA, Molecular Probes) or 0.1% DMSO (control) in KRH buffer for 30 min at 37 °C with constant shaking in the dark. Subsequently, hepatocytes were washed once with KRH buffer and 2'-7'-dichlorodihydrofluorescein (DCF) fluorescence was detected by a spectrophotometer plate reader (Molecular Devices, SpectraMax M5, Sunnyvale, CA, USA) at an excitation wavelength of 488 nm and an emission wavelength of 520 nm. DCFH-DA studies were conducted three times for each experimental group with 2 mice per group.

### 3.5. RNA Isolation, cDNA Synthesis and Real-time Quantitative PCR Analysis

Liver tissues washed with PBS were lysed in Trizol^®^ (Invitrogen Corporation, Grand Island, NY, USA) and total RNA was isolated according to the manufacturer’s instructions. cDNA synthesis and quantitative PCR were performed, as published previously [30]. Primers (sense: 5'-ACTCGGACCCAGGCTGC-3'; antisense: 5'-AGATAGGTGGTGCTGCTCAGG-3') and Taqman fluorescent probe (5' 6-[FAM]-TGTCTCCTGCTTCTCCTCCTTGCCA-3' [TAMRA-Q]) flanking about 70 base pairs of open reading frame sequences of mouse hepcidin genes, *Hamp1* and *Hamp2* were designed by the Primer Express 1.5 program (Applied Biosystems). ATF4 and ATF6 mRNA expression were determined by SYBR Green real-time PCR by using specific primers for ATF4 (sense: 5'-CCT GAA CAG CGA AGT GTT GG-3'; antisense: 5'-TGG AGA ACC CAT GAG GTT TCA A-3') and ATF6 (sense: 5'-TCG CCT TTT AGT CCG GTT CTT-3'; antisense: 5'-GGC TCC ATA GGT CTG ACT CC-3') mouse genes. Glyceraldehyde-3-phosphate dehydrogenase (gapdh) gene was used as the endogenous control (sense: 5'-GTGGAGATTGTTGCCATCAACGA -3'; antisense: 5'-CCCATTCTCGGCCTTGACTGT-3').

### 3.6. Analysis of X-Box Binding Protein 1 Splicing

ER stress-induced splicing of X-box binding protein 1 (XBP1) was determined by RT-PCR and restriction enzyme digestion. cDNA synthesized from mice liver RNA were employed to amplify XBP1 region containing inositol-requiring enzyme 1 (IRE1) excision site using specific primers (sense: 5'-AAACAGAGTAGCAGCGCAGACTGC-3'; antisense: 5'-TCCTTCTGGGTAGACCTCTGGGAA-3'). Amplified DNA was subsequently digested with Pst1 enzyme at 37 °C for 2 h and resolved on a 2% agarose gel, which was stained with ethidium bromide to detect XBP1 DNA fragments. The splicing experiments were repeated three times.

### 3.7. Chromatin Immunoprecipitation (CHIP)

CHIP assays were performed, as described previously [42]. Briefly, sheared chromatin were immunoprecipitated by using commercial antibodies (Cell Signaling, Santa Cruz, CA, USA). An aliquot of pre-cleared chromatin was saved as total input DNA prior to the immunoprecipitation. Eluted DNA were analyzed by PCR using primers specific for mouse *Hamp1* promoter (sense: 5'-GCCATACTGAAGGCACTGA-3'; antisense: 5'-GTGTGGTGGCTGTCTAGG-3'). CHIP assays were repeated three times with different sets of sheared chromatin.

### 3.8. Western Blotting

Preparation of total or nuclear cell lysates and western blots were performed, as described previously [42]. Each blot was repeated three times with different lysate preparations. All antibodies were obtained commercially (Cell Signaling, Santa Cruz).

### 3.9. Statistical Analysis

SPSS software was used for statistical analysis. The significance of difference between groups was determined by Student’s t-test, and non*-*parametric Kruskal-Wallis ANOVA and Wilcoxon Mann-Whitney tests. A value of *p* < 0.05 was accepted as statistically significant.

## 4. Conclusions

Hepcidin, synthesized primarily by the liver, protects the body from the harmful effects of iron by tightly controlling iron uptake and release. We have previously demonstrated that alcohol-induced oxidative stress suppresses liver hepcidin expression and thereby elevates intestinal iron transporter expression. A role for superoxide free radicals has been excluded in this process [29]. In this study, we investigated the role of H_2_O_2_ in hepcidin regulation by alcohol by using two different knockout mouse models with defective H_2_O_2_ catabolism. Comparison of catalase and gpx-1 knockout mice suggested that H_2_O_2_ inhibits hepcidin expression in the liver *in vivo*. In synergy with alcohol and in the absence of gpx-1, H_2_O_2_ induced ER stress via CHOP, independent of C/EBPα or CREBH, and thereby up-regulated hepcidin expression (Scheme 1)_._ These studies will help us to further understand the role of iron and redox metabolism in the pathogenesis of alcoholic liver disease.
